# 

**Published:** 1887-11-05

**Authors:** 


					f"- No.
jTT
58, Vol. III.
November 5th, 1887.
[Registered at the General Post Office
as a Newspaper.]
CHIlit o N fc. rtwn i.
0
Per Annum, 6s. 6d., post free.
Monthly Parts, 6d.; post free, 7id.
Ditto per Ann., 7s. 6d , ycst free.
Journal of
Science, flftebidne, anb ft>btlantbvop\>.

				

